# Cardiac and renal dysfunction is associated with progressive hearing loss in patients with Fabry disease

**DOI:** 10.1371/journal.pone.0188103

**Published:** 2017-11-21

**Authors:** Maria Köping, Wafaa Shehata-Dieler, Mario Cebulla, Kristen Rak, Daniel Oder, Jonas Müntze, Peter Nordbeck, Christoph Wanner, Rudolf Hagen, Sebastian Schraven

**Affiliations:** 1 Department of Oto-Rhino-Laryngology, Plastic, Aesthetic and Reconstructive Head and Neck Surgery, Comprehensive Hearing Center, University Hospital Würzburg, Würzburg, Germany; 2 Department of Internal Medicine I, Comprehensive Heart Failure Center (CHFC) and Fabry Center for Interdisciplinary Therapy (FAZIT), University Hospital Würzburg, Würzburg, Germany; University of Palermo, ITALY

## Abstract

**Background:**

Fabry disease (FD) is an X-linked recessive hereditary lysosomal storage disorder which results in the accumulation of globotriaosylceramid (Gb3) in tissues of kidney and heart as well as central and peripheral nervous system.

Besides prominent renal and cardiac organ involvement, cochlear symptoms like high-frequency hearing loss and tinnitus are frequently found with yet no comprehensive data available in the literature.

**Objective:**

To examine hearing loss in patients with FD depending on cardiac and renal function.

**Material and methods:**

Single-center study with 68 FD patients enrolled between 2012 and 2016 at the Department of Oto-Rhino-Laryngology, Plastic, Aesthetic and Reconstructive Head and Neck Surgery of the University of Würzburg. Every subject underwent an oto-rhino-laryngological examination as well as behavioral, electrophysiological and electroacoustical audiological testing. High-frequency thresholds were evaluated by using a modified PTA_6_ (0.5, 1, 2, 4, 6, 8) and HF-PTA (6, 8 kHz). Renal function was measured by eGFR, cardiac impairment was graduated by NYHA class.

**Results:**

Sensorineural hearing loss was detected in 58.8% of the cohort, which occurred typically in sudden episodes and affected especially high frequencies. Hearing loss is asymmetric, beginning unilaterally and affecting the contralateral ear later. Tinnitus was reported by 41.2%. Renal and cardiac impairment influenced the severity of hearing loss (*p* < 0.05).

**Conclusions:**

High frequency hearing loss is a common problem in patients with FD. Although not life-threatening, it can seriously reduce quality of life and should be taken into account in diagnosis and therapy. Optimized extensive hearing assessment including higher frequency thresholds should be used.

## Introduction

Fabry disease (FD) is an X-linked lysosomal storage disorder which causes the accumulation of globotriaosylceramid (Gb3) in different tissues due to a deficiency or absence of the lysosomal hydrolase α-galactosidase A [1, 2, 3, 4]. The incidence was previously reported to be between 1:40 000 to 1:117 000 [5, 6], however recent studies indicate higher occurrences of pathogenic mutations with demographic and ethnical impact [7, 8]. Both sexes can be affected with reduced life expectancy, whereby hemizygous males are affected more seriously than heterozygous women [9]. This accumulation of Gb3 affects kidney, heart as well as central and peripheral nervous system [10]. Clinical symptoms range from progressive kidney disease and cardiomyopathy to cerebrovascular complications including neuropathic pain and stroke [3, 4]. Furthermore, the vestibulocochlear system can be involved including a progressive hearing loss, which is typically asymmetric and occurs in episodes, as well as vestibular dysfunction with vertigo [11, 12, 13].

In a histological temporal bone study of two male patients with FD Schachern et al. [14] reported on a hyperplastic mucosa and seropurulent effusion in the middle ear as well as strial and spiral ligament atrophy and loss of outer hair cells. A storage of glycosphingolipid could not be found.

Since 2001, enzyme replacement therapy (ERT) with two recombinant enzyme products (agalsidase alfa, Replagal®, Shire, Lexington, MA, USA; agalsidase beta, Fabrazyme®, Genzyme Sanofi, Cambridge, MA, USA) is available [15, 16]. This treatment has been shown to enable a reduction of the storage of Gb3 in the kidneys [17] and reduce the left ventricular hypertrophy [18].

Due to the severity of the organ failure of kidneys, heart and brain, other symptoms like hearing loss and tinnitus are neglected even if they induce a serious reduction of the quality of life in patients with FD. The aim of this study was to examine the incidence of hearing loss and its correlation to renal and cardiac involvement in patients suffering from FD.

## Material and methods

### Subjects

Between 04/2012 and 11/2016, 68 consecutive FD patients (31 male, 37 female; 45.9 +/- 13.8 years; range 19–77 years) who attended the Department of Oto-Rhino-Laryngology, Plastic, Aesthetic and Reconstructive Head and Neck Surgery in Würzburg were comprehensively investigated after informed oral and written consent had been obtained appropriate to the decision of the institutional review board of the medical department Würzburg (220/15_z) which approved this study. All these patients were cases treated at the Würzburg Fabry Center for Interdisciplinary Therapy (FAZIT) within the scope of routine check-ups (that take place in intervals of 1 to 4 years) irrespective of possible ENT symptoms or comorbidities. Therefore, we can exclude preselection bias. Patients who presented for first contact in FAZIT were not enrolled. Further inclusion criteria were age ≥ 18 years and confirmed diagnosis of FD. This was achieved in each patient by DNA testing and performing an α-galactosidase A assay. Since the initiation of FAZIT in 2001 over 270 FD patients have been registered up to now.

### Clinical examination

All patients underwent a full oto-rhino-laryngological examination after medical history was taken. Otological data were collected, including hearing impairment, tinnitus and history of sudden hearing loss. In addition, we selectively enquired about possible confounders like infections (zoster oticus, morbilli, mumps, meningitis etc.), ototoxic medication (antibiotics, chemotherapeutics etc.) or noise exposure.

The audiological testing consisted of behavioral tests, such as pure tone audiometry and Freiburger monosyllables speech test, as well as electrophysiological and electroacoustical tests, such as otoacoustic emissions (OAE), auditory brainstem response audiometry (ABR) and tympanometry. Renal function was evaluated by measuring the glomerular filtration rate (estimated by the CKD-EPI equation), which was graduated as follows: ≥ 90, 60–89, 30–59 and ≤ 29 ml/min/1.73 m^2^ [19]. Cardiac function in patients with cardiac disease was classified by the NYHA score (class 1: no limitation of physical activity; class 2: slight limitation of physical activity, ordinary physical activity results in fatigue, palpitation or dyspnea; class 3: marked limitation of physical activity, comfortable at rest, less than ordinary activity causes fatigue, palpitation or dyspnea; class 4: unable to carry out any physical activity without discomfort, symptoms of heart failure at rest) [20]. Patients without any structural cardiac disease were admitted to class 0.

## Audiological measurements

All audiological measurements were performed with calibrated instruments in a sound-proofed room (DIN EN ISO 8253). The audiological evaluation included standard pure-tone audiometry (air conduction AC: 0.25 through 8 kHz; bone conduction BC: 0.5 through 6 kHz), conducted with a clinical audiometer in 5-dB steps. Hearing thresholds were then averaged in standard 4-pure tone average (PTA_4_: 0.5, 1, 2, 4 kHz), summarizing all values and dividing by 4 so every threshold carries equal weight. Furthermore, a modified PTA_6_ (0.5, 1, 2, 4, 6, 8 kHz) and high-frequency pure tone average (HF-PTA: 6, 8 kHz) were used to include the high-frequency range. HF-PTA has been established by Kuemmerle-Deschner et al. for diagnosis of another rare disease before [21]. Values were considered abnormal by difference of ≥ 10 dB from normative hearing thresholds (calculated based on [22]).

Functional disability due to hearing loss, as defined by the World Health Organization WHO by identifying standard PTA_4_ of the patient’s better ear, was described: grade 0: no impairment (PTA_4_ ≤ 25 dB), grade 1: slight impairment (PTA_4_ 26–40 dB), grade 2: moderate impairment (PTA_4_ 41–60 dB), grade 3: severe impairment (PTA_4_ 61–80 dB) and grade 4: profound impairment (PTA_4_ ≥ 81 dB). [23]

Speech discrimination was tested in quiet (“Freiburger monosyllables speech test”) at 65 dB and 80 dB SPL in 65/68 patients.

Otoacoustic emissions (Etymotic ER10, Illinois, USA) as well as the tympanometry (MT10 tympanometer, Interacoustics, Middelfart, Denmark) were performed in each patient.

Furthermore, ABR was performed using a clinical evoked potential measurement system Eclipse—ASSR EP15/EP25 (Interacoustics, Middelfart, Denmark) in 63/68 patients. Click stimuli were presented to the individuals at intensities between 10 and 100 dB HL. The responses were then averaged and the ABR threshold was visually determined where wave V showed the smallest response amplitude.

### Statistical significance

Shapiro-Wilk test did not demonstrate a normal distribution, so Kruskal-Wallis test and Wilcoxon’s rank-sign test for matched pairs were performed. Statistical significance was set at the 95% confidence level and above (*p* < 0.05). Error bars indicate standard deviation.

## Results

All 68 FD patients included showed normal otoscopy examination results. Of all patients 32.4% complained about hearing loss; unilateral hearing loss was described by 6 male and 5 female, whereas bilateral hearing loss was reported by 11 male patients. All affected patients reported on sudden episodes of hearing loss, beginning unilaterally and in some cases affecting the contralateral ear later. Tinnitus was described by 28 patients (male 16, female 12). Aside from information of 3 patients (2 patients wearing hearing protection because of noise exposure at work, 1 man with history of an acute acoustic trauma), no noticeable risks concerning inner ear damage could be revealed. Thirty-one persons did not have any of the symptoms named above. At the date of examination 41 patients received ERT (male 24, female 17), 3 men had interrupted therapy prior due to intolerance. The mean age at onset of ERT was 43.9 years (range 12–73 years) with a mean period of time on medication of 5.73 years (range 1–12 years).

Renal function of every patient was determined with the eGFR and grouped according to KDIGO categories G1-4. Twenty-six subjects (male 13, female 13) showed a value of ≥ 90, 23 patients (male 8, female 15) a value of 60–89, 15 patients (male 6, female 9) a value between 30–59 and 4 male patients a value of ≤ 29 ml/min/1.73 m^2^. All subjects were also grouped in 4 classes according to the NYHA score. 26 subjects (male 13, female 13) were scored within class 0, 15 subjects (male 9, female 6) in class 1, 19 patients (male 6, female 13) in class 2 and 8 patients (male 3, female 5) in class 3 (**Table 1**).

**Table 1:** Main clinical symptoms and characteristics of all 68 patients.

	*male*	*female*	*total (%)*
***Patients*** *(n)*	31	37	68 (100)
***Mean Age / Range***	41.9 (19–75)	49.3 (24–77)	45.9 (19–77)
***Case history***			
Hearing loss	17	5	22 (32.4)
- unilateral	6	5	11 (16.2)
- bilateral	11	0	11 (16.2)
Tinnitus	16	12	28 (41.2)
Asymptomatic	9	22	31 (45.6)
***ERT***	27	17	44 (64.7)
Current	24	17	41 (60.3)
Aborted	3	0	3 (4.4)
***GFR*** *(ml/min/1*.*73m*^*2*^*)*			
≥ 90	13	13	26 (38.2)
60–89	8	15	23 (33.8)
30–59	6	9	15 (22.1)
≤ 29	4	0	4 (5.9)
***NYHA Class***			
0	13	13	26 (38.2)
1	9	6	15 (22.1)
2	6	13	19 (27.9)
3	3	5	8 (11.8)

ERT: enzyme replacement therapy.

**Fig 1:**
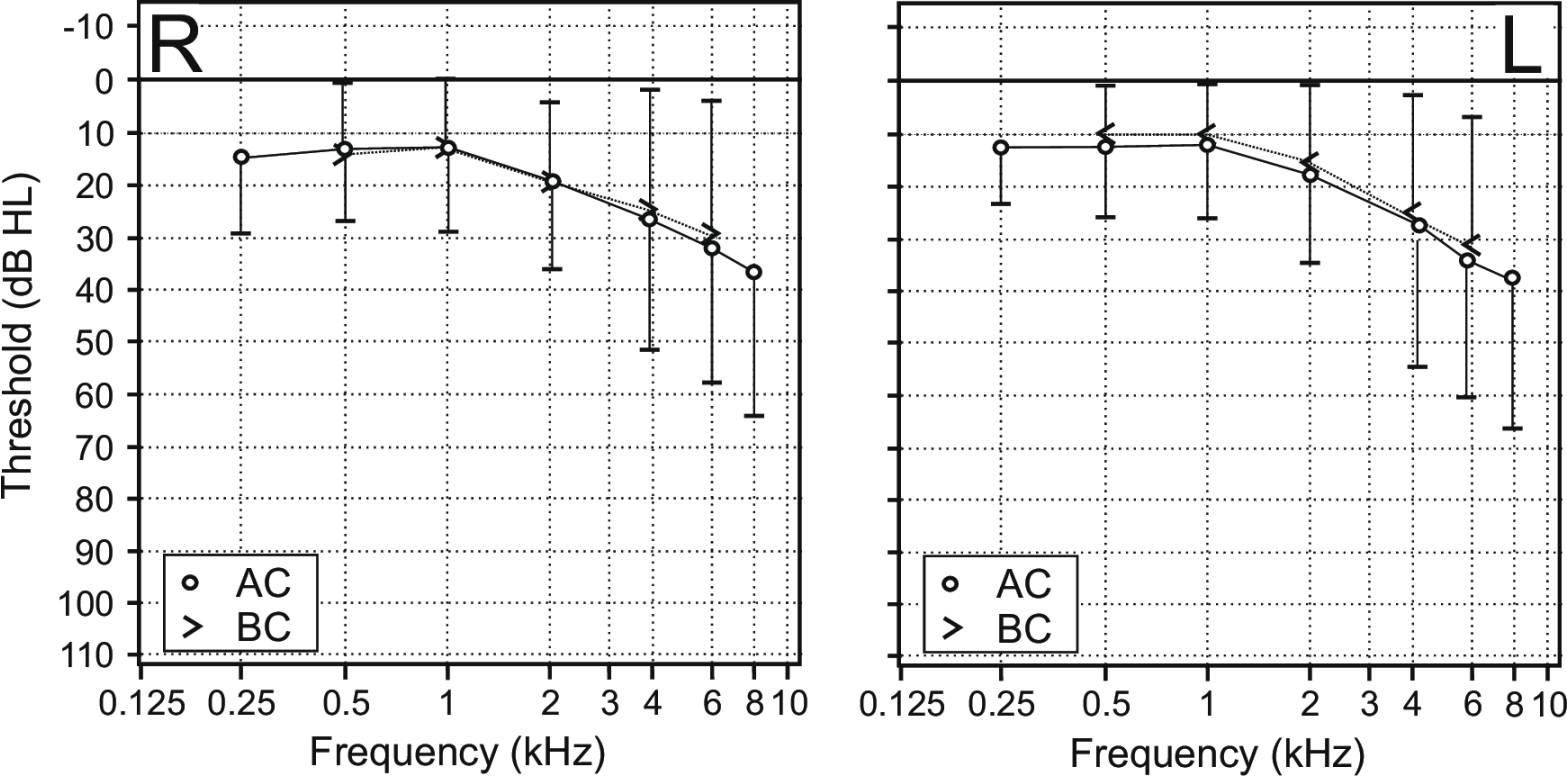
Mean pure tone audiometry thresholds show a high frequency sensorineural hearing loss measured for each right (R) and left (L) ear (n = 68). Air conduction (AC, circles), bone conduction (BC, triangle). Error bars depict standard deviation.

Pure-tone audiometry was performed in all 68 patients measuring air and bone conduction of both ears. None of the patients offered conductive or combined hearing loss, so that in further it is only focused on AC. Although only 32% of the patients complained about uni- or bilateral reduced hearing, a sensorineural hearing loss ≥ 25 dB HL was observed in 59% (25 male and 15 female) of the individuals (**Fig 1**). Men were affected more severely than women.

All patients showed a type A tympanogram indicating a normal middle ear pressure and normal mobility of the tympano-ossicular chain. The OAE showed an averaged reproducibility of 91.6% in the right and 89.2% in the left ear (SD 14.0 resp. 19.5). The values of OAE were consistent with the findings in pure tone and speech audiometry and confirmed the degree of hearing loss.

**Fig 2:**
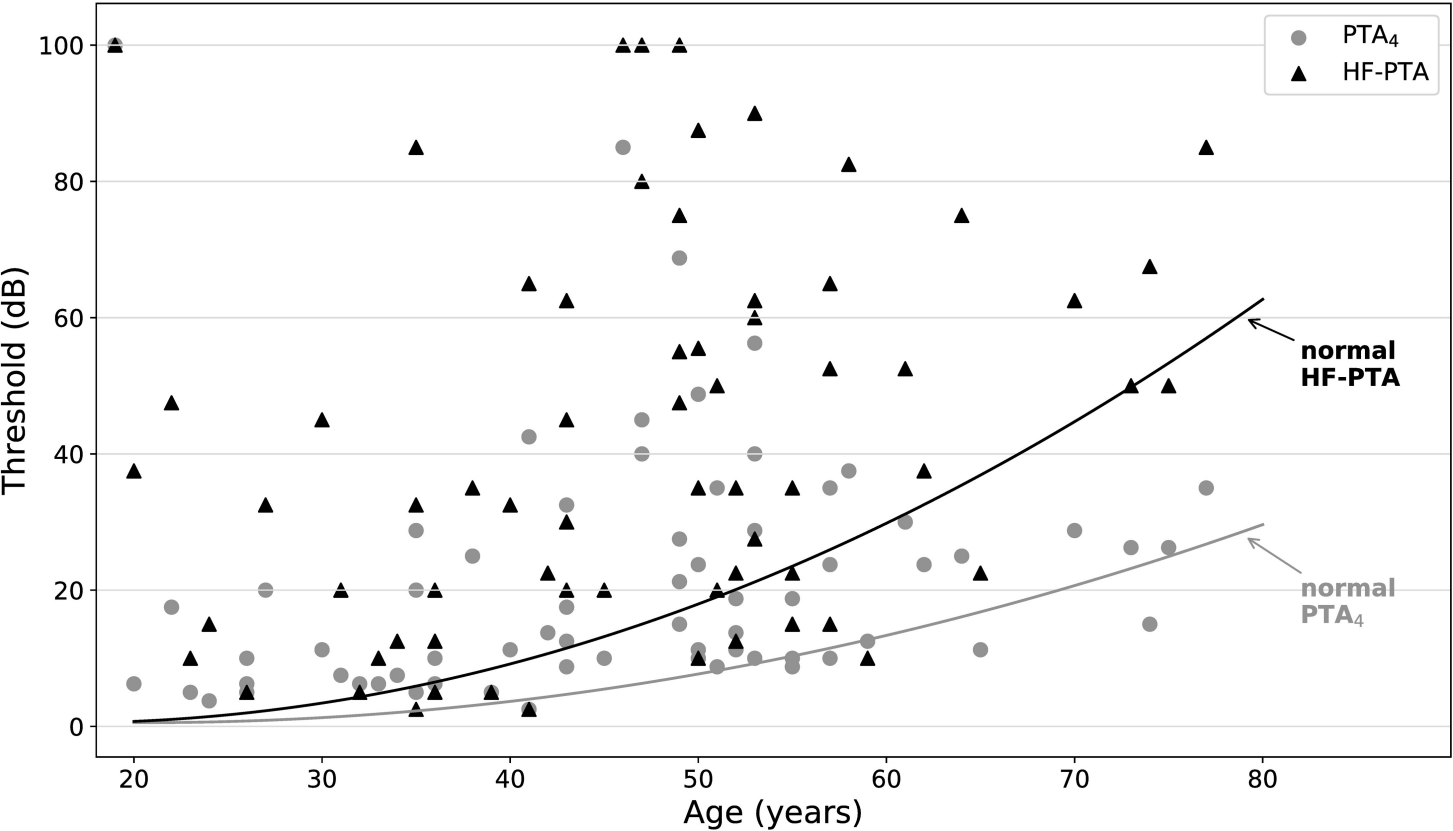
Averaged pure tone thresholds PTA_4_ and HF-PTA of all FD patients (n = 68) compared to age specific median thresholds of healthy controls. Normal thresholds (HF-PTA: black line, PTA_4_: grey line) decline with age. After adjusting for age, FD patients show highly increased thresholds in PTA_4_ (grey circles) and significantly increased thresholds in HF-PTA (black triangles).

Respecting the age specific median limits of hearing thresholds (calculated from [21] and [22]) 26/68 FD patients (38.2%) had abnormal PTA_4_ values, the mean absolute deviation from the standard curve was 14.7 dB. The median PTA_4_ was 26.6 dB in men and 17.4 dB in women. 41/68 patients (60.3%) had abnormal HF-PTA values, mean deviation from the standard curve being 26.4 dB. The median HF-PTA was 51.3 dB in men and 32.0 dB in women (**Fig 2**).

According to the WHO classification of disability due to hearing loss measured by PTA_4_ of the good ear, only 7 patients (10.3%) showed pathological values with slight and moderate impairment (mean PTA_4_ value 13.6 dB, range 2.5–53.8). Looking at the bad ear 21 patients (30.9%) were found with at least slight impairment (mean 21.6 dB, range 2.5–100).

**Fig 3:**
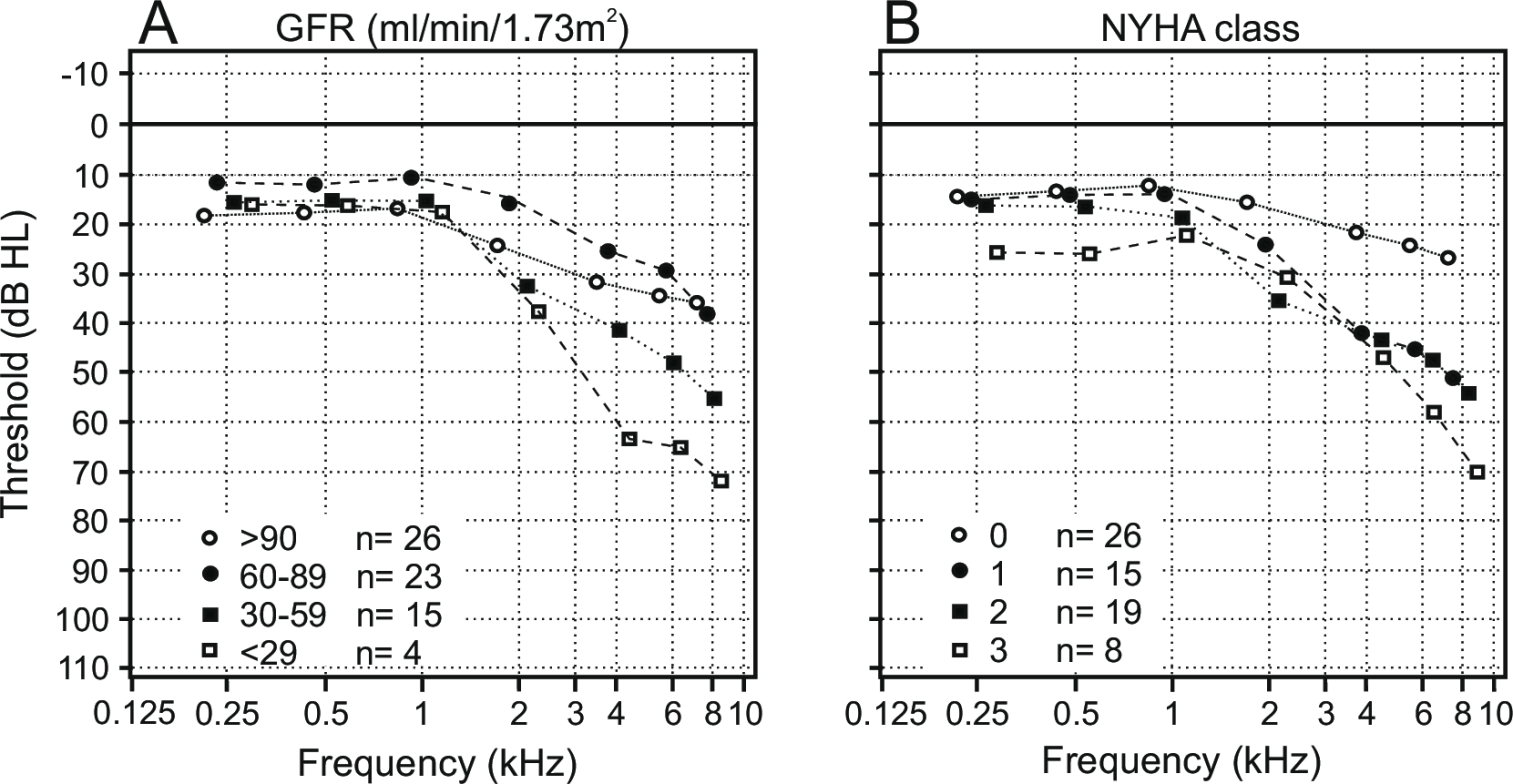
Air conduction pure tone thresholds of the bad ear (n = 68) in relation to renal (**A**) and cardiac (**B**) function. A pronounced increase of hearing threshold at higher frequencies is shown, depending on the severity of renal function, evaluated by the GFR (ml/min/1.73 m^2^) and graduated in four groups (≥90, 60–89, 30–59 and ≤ 29) and cardiac function, classified by the NYHA class (0, 1, 2, 3).

Subgroups of renal loss of function (GFR class; **Fig 3A**) and cardiac impairment (NYHA class; **Fig 3B**) showed no significant difference at low- and mid-frequency range (0.25 kHz to 2 kHz). At higher frequencies, a steep slope of hearing thresholds depending on the severity of renal and cardiac function was observed.

**Fig 4:**
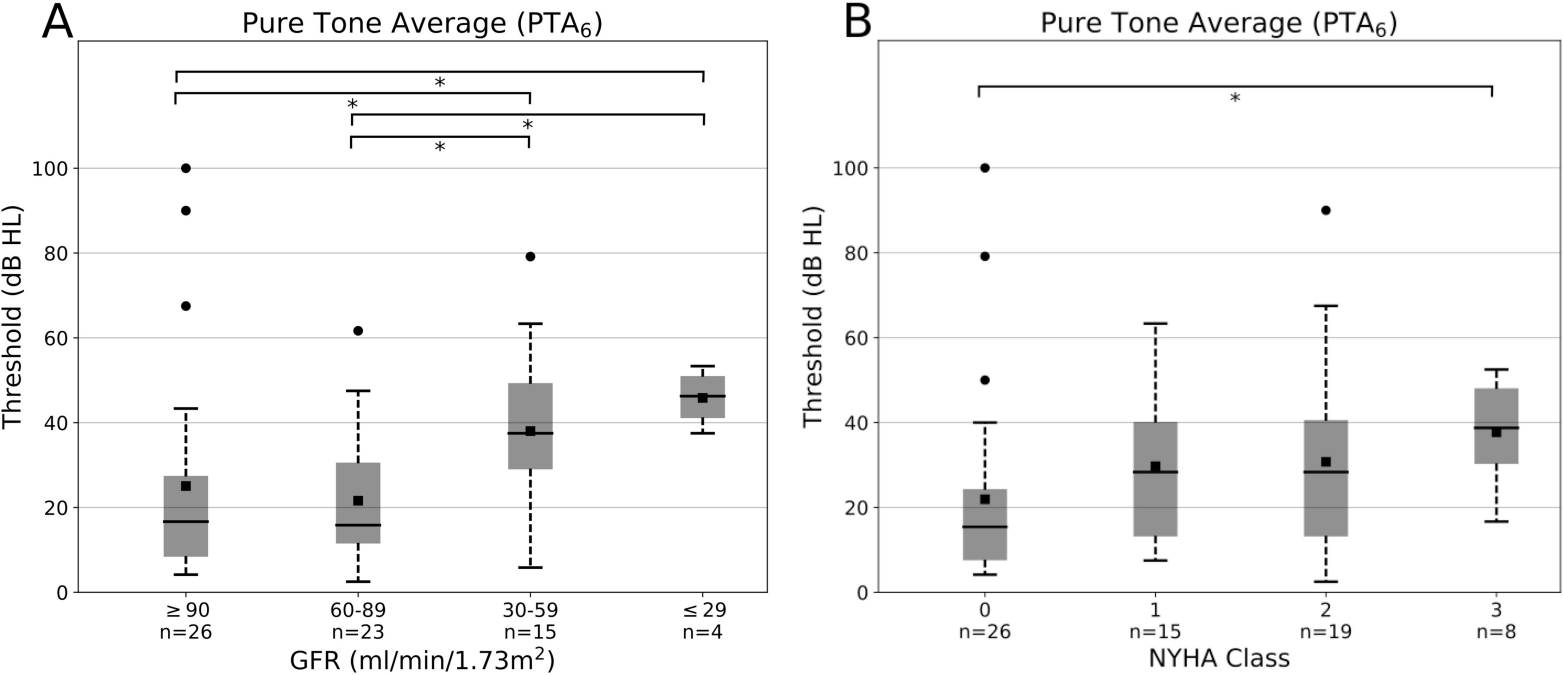
Mean PTA_6_ (0.5, 1, 2, 4, 6, 8 kHz) AC thresholds for the bad ear (n = 68) in correlation to (**A**) GFR (ml/min/1.73 m^2^, graduated in four groups: ≥90, 60–89, 30–59 and ≤ 29) and (**B**) NYHA class (0, 1, 2, 3). Asterisks indicate p<0.05. Error bars depict standard deviation.

This phenomenon is also reflected in pure tone average PTA_4_ and modified PTA_6_. While the results in PTA_4_ were less significant, the high frequency hearing loss was better depicted by PTA_6_ showing a mean value of 34.8 dB in men and 22.2 dB in women (SD 22.8 resp. 17.5) in contrast to PTA_4_ with levels between 26.6 dB in men and 17.4 dB in women (SD 21.0 resp. 14.9) as already described above. Here again, a significantly increasing hearing loss could be seen in relation to NYHA class and GFR (**Fig 4A and 4B**, showing only PTA_6_).

**Fig 5:**
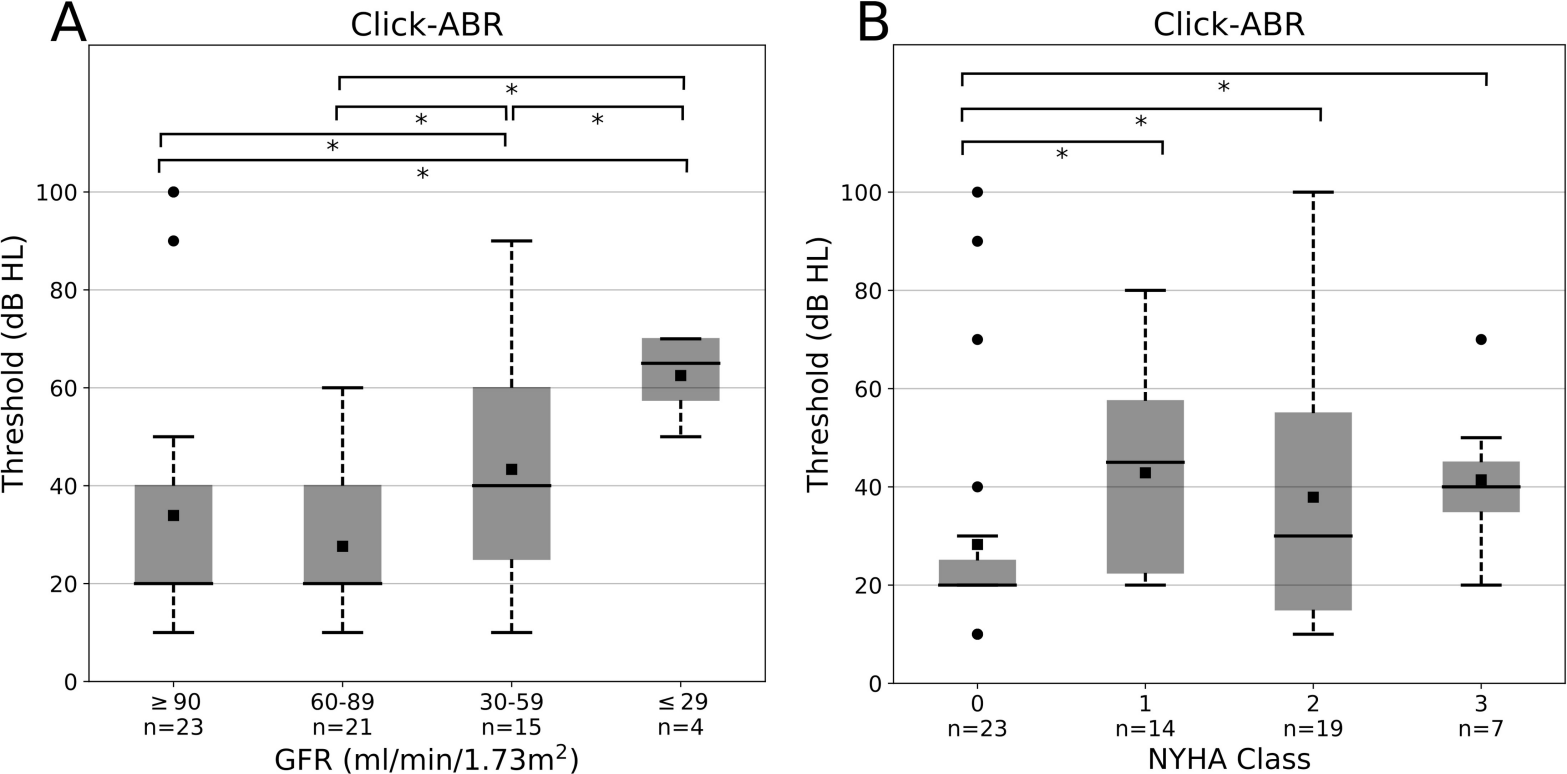
Click-ABR of the patient’s bad ear (n = 63) according to (**A**) renal and (**B**) cardiac function. Renal function was evaluated by GFR (ml/min/1.73 m^2^) and graduated in four groups (≥90, 60–89, 30–59 and ≤ 29). Cardiac function was classified by NYHA class (0, 1, 2, 3). Asterisks indicate p<0.05. Error bars depict standard deviation.

Click-ABR analysis revealed normal interpeak latencies I-III, III-V and I-V in all patients, so retrocochlear lesions could be excluded. Referring to GFR values ABR thresholds showed a significant increase at decreasing kidney function. This could also be seen at rising NYHA class (**Fig 5A and 5B**).

**Fig 6:**
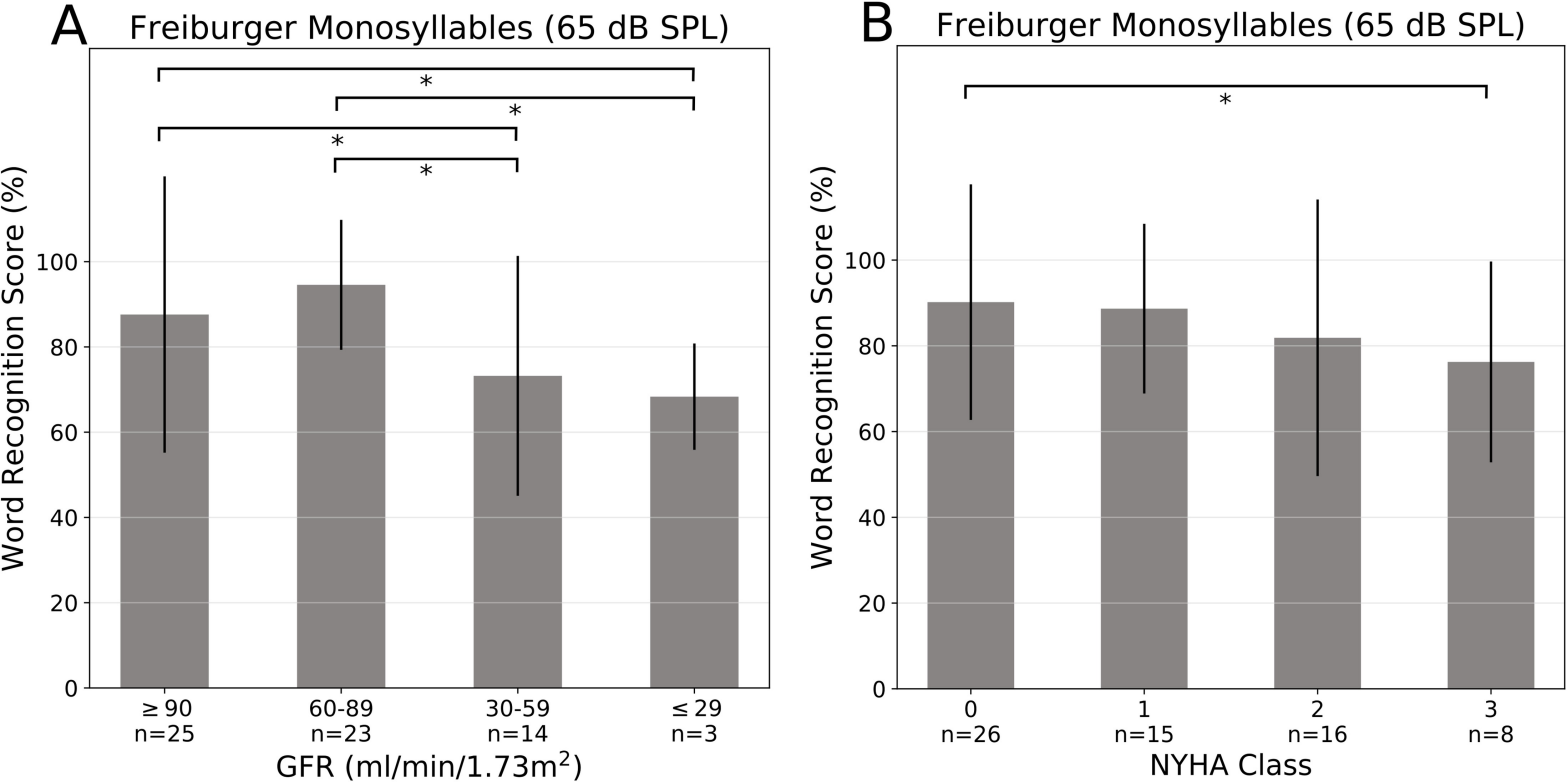
Mean speech audiometry results in quiet (“Freiburger monosyllables speech test” at 65 dB SPL). Only the results of the bad ear (n = 65) are shown. (**A**) Renal function evaluated by the GFR (ml/min/1.73 m^2^) and graduated in four groups (≥90, 60–89, 30–59 and ≤ 29). (**B**) Cardiac function was classified by the NYHA score (0, 1, 2, 3). Asterisks indicate p<0.05. Error bars depict standard deviation.

Speech audiometry in quiet conditions (Freiburger monosyllables test) at 65 dB SPL (average conversational intensity level) showed a significant impairment of word recognition scores with decreasing renal function (**Fig 6A**) and increasing NYHA class (**Fig 6B**). At 80 dB SPL the differences between the groups were less (not pictured). The scores were within the expected range compared to pure tone audiometry thresholds.

## Discussion

The results of the current single-center study demonstrate that hearing impairment is common in FD and that the severity of renal and cardiac organ involvement is significantly associated with the grade of hearing loss.

This is the first systematic evaluation of hearing loss in FD including behavioral, electrophysiological and electroacoustical measurements in regard to renal and cardiac function.

In the current group of 68 FD patients a high incidence (58.8%) of sensorineural hearing loss could be found, affecting mainly the high frequency range and occurring typically in sudden episodes. In contrast to other publications like Hegemann [24], who had a selected patient cohort regarding FD patients with ear-related symptoms and before initiation of ERT only, we excluded preselection with the objective of identify the prevalence of cochlear disorders in the whole FD group. Most of previous reports examined audiological findings in smaller case series, Germain, Conti and MacDermot could also show progressive sensorineural hearing loss [11, 12, 9]. Two larger case series exist with 86 [24] and 72 [25] mixed-gender patients, also depicting hearing loss in approximately 50% of cases and affecting hemizygous men more severely than heterozygous women: Limberger [25] found an accentuation in higher frequencies, whereas Hegemann [24] could not confirm these findings. A possible reason for the discrepancy could be that Hegemann had focused on severely affected people which already had symptoms. Another aspect would be that ERT has a positive influence on the hearing threshold.

In the age-matched examination of PTA_4_ and HF-PTA we saw higher threshold levels in FD patients than in the control group. Doubtless, what should be kept in mind is that there could be a wealth of other reasons for high-frequency hearing loss, but the confounding age-related presbyacusis can be eliminated. Further could be criticized that GFR and NYHA class could worsen in old age. In future work, the generation of age- and gender-related control groups for GFR resp. NYHA class is planned.

The results are very promising but show that some categories of GFR and NYHA could only be covered by few cases and prevent a more detailed evaluation (see also S1 File). So, it is planned to continue data collection to allow reliable age and gender dependent statistical analysis in future.

In the current study, predominantly a high-frequency sensorineural hearing loss was found. No conductive or mixed hearing loss was detected, all patients had a normal middle ear ventilation represented by type A tympanogram. Since retrocochlear pathology was excluded by ABR, the assumption that the lesion is located in the inner ear is emphasized [11, 12, 26]. This is confirmed by the histological results of Schachern et al., who described morphologically regular ganglion cells, which were reduced in the basal turn of the cochlea, as well as an atrophic spiral ligament and stria vascularis. However, an accumulation of Gb3 was not found [14]. Nevertheless, a vascular damage, caused by lysosomal storage of Gb3 in endothelial cells or smooth muscle cell proliferation with consecutive infarction of primarily small vessels, should also be considered [1]. An accumulation in podozytes and cardiomyozytes could already be demonstrated [27]. Further anatomic studies are essential to distinguish the pathophysiological source of hearing loss.

Germain et al. found a significant increase of high frequency hearing impairment with kidney failure in a group of 22 hemizygous male patients, out of which 12 subjects already had severe or end-stage kidney disease. A correlation with left ventricular hypertrophy could not be demonstrated [11]. In our cohort patients were healthier; chronic kidney disease (CKD) with an eGFR ≤ 29 ml/min/1.73m^2^ occurred in only 4 patients (5.9%). But again, a significant aggravation of hearing threshold, speech perception and ABR thresholds with decreasing kidney function could be shown. This goes along with Vibert’s hypothesis that cochleovestibular symptoms correlate with CKD of every stage [28].

In regard to the fact that hearing loss in patients with FD is mainly limited to higher frequencies, the PTA_4_ value is not suitable for a correct classification. In 2003, Conti assumed that hearing loss was underestimated in standard PTA (0.5, 1, 2 kHz) [12]. In the modified PTA_6_ value frequencies above 2 kHz are also included. Thus, in this study significantly higher values were found in PTA_6_ compared to PTA_4_. Another option is to focus exclusively on higher frequencies as done by HF-PTA. In addition to standard audiological measurements, enclosing behavioral, electrophysiological and electroacoustical testing, an optimized evaluation including high frequency thresholds in FD patients is recommended. Even if standard testing shows normal values, high frequency hearing impairment can occur and cause problems such as reduced word discrimination.

Up to now, it is not yet proven whether ERT can ameliorate hearing loss. Hajoff could show a gradual improvement at 4 and 6 kHz under agalsidase alfa in a group of 15 hemizygous males [29]. Palla et al. reported on a significantly higher incidence of hearing loss in male patients as well as at higher ages, but could not reveal a significant change of hearing thresholds in a mixed-gender group of 38 patients after 60 months of ERT [13]. In two small mixed-gender groups of 20 respectively 14 FD patients Sergi as well as Conti supposed that ERT could stabilize hearing function [30, 12]. In our cohort, the mean age at onset of therapy was 43.9 years. Further studies will investigate the benefit of an earlier onset of ERT. Cochleovestibular symptoms besides other Fabry-specific symptoms like limited performance and acroparesthesia can strongly impact quality of life. In conclusion, there is a need to include cochlear symptoms in decision-making on initiation of Fabry-specific therapy.

## Conclusion

High-frequency hearing loss is a clinically relevant complication in FD. The severity of renal and cardiac impairment has been found to significantly correlate with the grade of inner ear involvement. As this might tremendously affect patients’ quality of life, hearing loss should be taken into account when evaluating the initiation of Fabry-specific therapy. Therefore, an optimized and extensive hearing assessment including higher frequency thresholds should be regularly applied in FD patients.

## Supporting information

S1 File Comparision PTA_4_ and GFR. Version 1: Gender unmatched, Version 2: Male, Female.
(PDF)

## Abbreviations

ABR: auditory brainstem response
AC: air conduction
BC: bone conduction
ERT: enzyme replacement therapy
FAZIT: Würzburg Fabry Center for Interdisciplinary Therapy
FD: Fabry disease
Gb3: globotriaosylceramid
GFR: glomerular filtration rate
NYHA: New York Heart Association
OAE: otoacoustic emission
PTA: pure tone average
WHO: World Health Organization
