# Supplementary material for: Cardiac and renal dysfunction is associated with progressive hearing loss in patients with Fabry disease
Source: PLoS One. 2017 Nov 21;12(11):e0188103. doi: 10.1371/journal.pone.0188103 (PMC5697846; doi:10.1371/journal.pone.0188103)

Comparision PTA_4 and GFR

Version 1: Gender unmatched

Version 2: Male, Female but male class "D" is lost because it doesn't exist in female

1)Male & Female

```
A  B  C  D (4 classes)
52 46 30  8

Kruskal-Wallis chi-squared
p-value = 1.248e-06

Post-hoc
  A       B       C
B 0.51608 -       -
C 0.00047 0.00047 -
D 0.00140 0.00068 0.19809
```

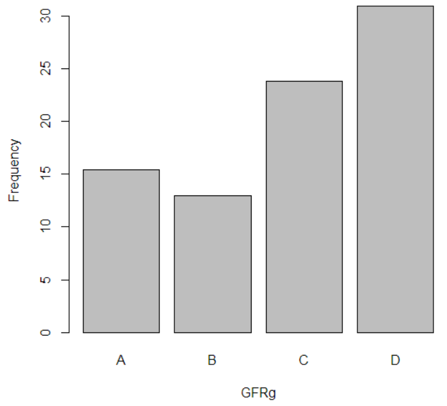

2a)Male, but without class “D” to meet females class distribution

```
A  B  C  (3 classes)
26 16 20

Kruskal-Wallis chi-squared
p-value = 0.004383

Post-hoc
  A      B
B 0.4913 -
C 0.0052 0.0417
```

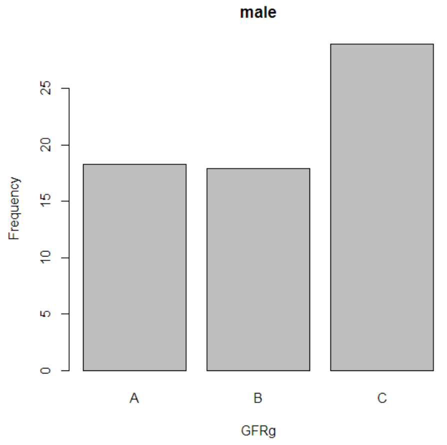

2b)Female

```
A  B  C  (3 classes)
26 30 18

Kruskal-Wallis chi-squared
p-value = 0.0001404

Post-hoc
  A       B
B 0.40910 -
C 0.00073 0.00035
```

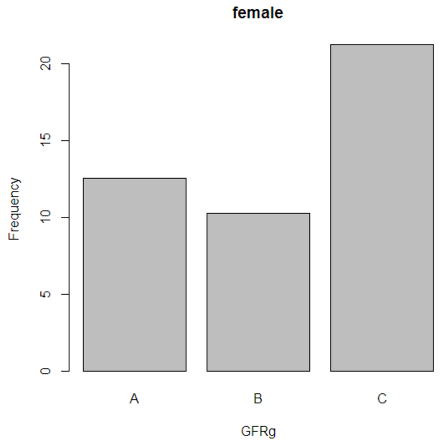

Supplement: S1 File — Version 1: Gender unmatched, Version 2: Male, Female. (PDF) [file pone.0188103.s001.pdf]
